# Supplementary material for: Are carbon emissions trading and green financial instruments synergistic? -Comprehensive quantitative research based on content analysis
Source: PLoS One. 2024 Mar 7;19(3):e0298601. doi: 10.1371/journal.pone.0298601 (PMC10919601; doi:10.1371/journal.pone.0298601)
Supplement: S1 Data — (ZIP) [file pone.0298601.s001.zip › Data/地级市Z_F工作报告.zip!/╕╩╦α/░╫╥°╩╨/└┤╫╘╣1⁄2╓┌║┼í╛┬φ┐╦╩2╛▌═°í┐_2015.docx]

各位代表：

现在，我代表市人民政府，向大会作工作报告，请予审议，并请政协委员及列席人员提出意见。

2014年工作回顾

2014年，在省委、省政府和市委的坚强领导下，我们深入贯彻党的十八大和十八届三中、四中全会精神，努力克服工业主导产品价格下跌、企业效益下滑和财政收支压力加大等实际困难，坚持稳中求进工作总基调，着力推进稳增长、调结构、促改革、惠民生各项工作，经济社会实现了平稳发展。全年完成生产总值497.58亿元，同比增长8.9%；规上工业增加值161.05亿元，增长10%；固定资产投资428.44亿元，增长21.71%；公共财政预算收入26亿元，增长7.83%；社会消费品零售总额152.35亿元，增长12.8%；居民消费价格上涨1.9%；城镇居民人均可支配收入20052.8元，增长9.7%；农民人均纯收入5777.4元，增长12.4%。

1.致力调整结构、培育产业，发展方式加快转变。三次产业结构由11.62:54.73:33.65调整为11.48:50.45:38.07，三产比重提高4.42个百分点，经济结构渐趋合理。

农业经济增产增效。粮食总产量突破80万吨，达到83万吨。新改扩建日光温室1.04万亩，瓜菜面积达到99.14万亩，实施“万吨农产品进京”工程，我市成为北京冬季蔬菜供应储备直供基地；生猪、牛、羊饲养量分别达到170.8万头、17.87万头和466.2万只，肉蛋奶总产量17.49万吨，规模化养殖比例达到78%，正大现代农牧等产业化项目建成运营；新增特色经济林14.75万亩。落实农机购置补贴资金3910万元，耕种收综合机械化水平达到49%。土地流转面积达到93.5万亩；农民专业合作社总数达到6130家，居全省首位；龙头企业总数达到343家，农产品加工转化率达到50%。推广全膜双垄沟播143.4万亩、高效节水示范灌溉94.1万亩，创建粮油高产万亩示范片20个，农作物良种覆盖率、畜禽良种普及率分别达到95%、90%以上。新增无公害农产品产地认定、产品认证33个，“靖远黑瓜籽”获得国家地理标志保护产品认证，“陇郁香”亚麻油、“御液香”梨荣获国家级农产品金奖，“高原宏”枸杞、“条山”早酥梨和苹果荣获国家级绿色食品金奖，靖远农业科技园升级为省级农业科技园。

工业经济转型提质。着力壮大“3+4”产业集群，60万吨尾矿制酸、凯宏建材生产线等项目建成投运，铜冶炼技术提升改造、锌冶炼资源综合利用、刘化集团年产25万吨硝基复合肥、恒大陶瓷等项目加快推进，电解铝液转化率达100%；加强工业运行调度，出台工业生产止滑稳增5条政策措施，28家企业进入全省直购电交易平台。全市煤炭、有色、电力、化工四大行业完成增加值123.7亿元，增长12.3%。积极培育战略性新兴产业，倚银石化装备生产基地建成投产，华电贾寨柯风电、北京万源五合风电、德祐光伏发电等项目建成并网，云计算产业园、500架无人机发动机和总装生产线、蓝通稀土高铁铝合金、碳纤维新材料、恒丰稀土多功能材料、尚德节能型电动机等项目顺利推进，稀土公司入选全省战新产业总体攻坚战首批骨干企业，全市规模以上战略性新兴产业完成增加值28亿元，增长21.6%，占GDP比重5.63%。白银高新区在全省35家工业园区综合考核中位列第三，白银工业集中区完成工业增加值150.4亿元，增长9.5%。

第三产业比重上升。银西工业园鼎丰物流一期工程投入使用，二期工程启动建设；中进大西北仓储物流园、靖远西北果蔬冷链物流中心动工建设；汽车商贸服务城26个4S店投入运营；国芳百货商贸综合体、义乌商贸城建成营运，金域观澜商贸综合体、靖远浙江商贸城等一批商贸综合体加快推进；新建和改扩建忠恒金地年鲜特色商品交易中心、白银粮油批发市场、五一街等农贸市场18个，农家超市260个；靖远县入选阿里巴巴“千县万村”计划，西北大磨坊、北方菜业等7家企业入驻“淘宝白银分馆”，电商企业达到41家，网络零售额增长26%。“新网工程”深入推进，恢复重建基层供销社7家。布设各类农村金融机构网点90个，便民服务点701个,创建信用村71个，发放双联惠农、妇女小额、牛羊蔬菜“三项贷款”17.47亿元，“三权”抵押贷款4606万元；全市金融机构存贷比71.15%，提高5.34个百分点，各项贷款余额407.31亿元，较年初增加60亿元，增长17.28%。怡园?靖远古城、会宁汉唐二十四节气文化商业街、白银区黄河假日城等文化旅游开发项目加快推进；黄河石林列入全省20个大景区建设规划，调整了景区管理体制；靖远腾飞国际赛车场和滑雪场建成投运，成功举办全国汽车场地越野锦标赛；会宁被评为“中国文化旅游名县”，景泰被评为“中国最具魅力文化旅游名县”。全市实现旅游收入32.93亿元,增长24.6%；完成文化产业增加值6.25亿元,增长24.05%；第三产业增加值增长7.1%。

循环经济成效显著。实施循环经济项目151项，完成投资91.2亿元；成功举办全省循环经济现场会、循环经济国际博览会，白银循环经济发展模式得到认可推广；“双百工程”示范基地加快推进，靖远被列为全省循环经济示范县，平川地膜回收利用列入国家农业清洁生产示范县建设项目；单位生产总值能耗和主要污染物排放提前完成“十二五”控制目标。

非公经济量质齐升。落实全省支持非公经济跨越发展的30项政策措施，拨付非公经济专项扶持资金3409万元，减免非公企业税收1.8亿元,新增非公经济主体1.64万户，比上年增长35.09%，总量突破7万户，非公经济投资占全市固定资产投资53.48%，增加值占GDP比重42.2%，纳税19.77亿元。“康视达”眼镜被评为中国驰名商标，“金杞福源”、“赛诺生物”等16件商标被评为甘肃省著名商标，商标有效注册量位列全省第三。

2.致力项目建设、加大投资，发展后劲持续增强。扎实开展“项目建设落实年”活动，全市在建项目860个，其中新开工项目608个，比去年增加252个。160个市列重点项目完成投资157.78亿元，21个项目建成投运。双永供水主干工程完工，引洮一期会宁北部供水工程实现通水，热电联产项目建筑主体基本完成，黄河石林二级公路、沿黄快速通道白银至水川连接线建成通车；凯斯瓷业高档墙地砖一期、中鑫石化清洁燃油等项目建成投运，晋江福源食品生产线、启明星高频无极灯二期扩建等重大项目进展顺利；矿田地质找矿取得新进展，新增铜铅锌金属量51.93万吨、金资源量10.23吨；亚行一期项目全面完成，总投资12.3亿元的亚行二期贷款项目完成谈判;干塘至武威南铁路增建二线、平堡黄河大桥及引线工程开工建设，包兰铁路扩能改造银川至兰州客运专线方案基本确定；引洮二期会宁供水工程项目建议书通过国家发改委批复，黄河白银段防洪治理项目可研通过水利部审查上报国家发改委待批，甘肃中部生态移民扶贫开发供水工程规划报告通过水利部水规总院审查。

3.致力深化改革、鼓励创新，发展活力不断释放。按照市委的统一部署，认真谋划和落实各项改革措施，实施创新驱动战略，科技创新对经济增长的贡献率达到50.5%。

简政放权力度加大。承接省上下放项目核准权12项、价格制定权11项。编制了行政审批事项目录，建立了权力清单和负面清单，取消下放审批事项165个，取消行政事业性收费5项。积极推进工商登记制度改革，将113项工商登记前置审批改为后置审批。财税金融改革深入实施。稳步推进预决算公开,出台了预算管理和政府债务管理等制度文件，扩大“营改增”试点范围。举办“银政企”项目对接会，落实贷款56.62亿元；新增农村资金互助组织87个；全市有106家中小微企业在上海、深圳、天津、甘肃等区域性股权交易市场挂牌，采取增资扩股、金融租赁、股权交易、私募债等方式直接融资13.41亿元。企业改革稳步推进。妥善解决原靖远陶瓷建材公司大集体企业长期未改制问题，核销养老保险金等费用2300万元；筹措资金8000万元，解决了长通集团改制职工医保等问题;按照“视同缴费”对未缴医疗保险过渡费的改制企业职工办理了退休手续。农村改革继续深化。建立和完善农村集体土地承包经营权数据库，完成11个乡镇74.6万亩土地的确权登记试点工作。科技创新取得突破。兰白科技创新改革试验区获科技部批准；白银科技企业孵化器被认定为国家级孵化器，一期生物医药中间体产业园、二期装备制造业产业园建成12.5万平方米，入孵企业100家，拥有专利149件；全市万人发明专利拥有量达到1.03件，位列全省第三，完成技术合同交易额2.5亿元；与上海张江、杨浦及部分企业建立了战略合作伙伴关系，与兰州理工大学联合建设“白银新材料研究院”，与兰州大学合作共建“白银技术转移中心”，与西北师范大学合作共建“白银专家服务基地”，引进高层次创新创业人才（团队）12个，新增高新技术企业4家、科技型企业13家、省级工程技术研究中心1家、省级重点实验室1家；白银原点科技公司晋级全国创新创业大赛总决赛，甘肃稀土新材料、长通电线电缆两家公司入选2014年国家火炬计划重点高新技术企业（全省3家）。对外合作不断加强。第20届兰洽会签约项目169个，开工率82.8%；与世界500强、国内500强、上市公司企业签约项目11个，签约资金284.3亿元；招商引资累计到位资金373亿元，同比增长35.87%。建立外贸促小育新项目库，申请贴息资金1774.2万元；全市实现进出口总额7.47亿美元。

4.致力夯实基础、统筹发展，城乡环境明显好转。加快城乡基础设施建设，提升城镇功能，全市城镇化率达44.93%，提高1.7个百分点。

城镇建设加速推进。城市总体规划、村镇规划编制完成；广场东路等3条道路和建设北路立交桥改造完成，银西中路、西山路南段等4条道路建设进展顺利；改造失管小区8个；城区新增绿地73.94公顷，成功争创“省级园林城市”；大力开展“五城联创、六城同建”，被确定为全国文明城市提名资格城市。实施城区防洪综合治理、污水处理再生利用和城区供水供热管网改造等工程,全年处理污水982.7万立方米，供水2286万吨，供热1326.75万平方米；启动实施中心街和八号楼什字旧城改造项目。购置环保型公交车41辆，新开通公交线路3条。靖远县、白银区四龙镇、会宁县郭城驿镇纳入全省新型城镇化试点范围。火焰山国家矿山公园建成运行。侯川、柴门、刘川、北滩、正路、中泉、宝积撤乡改镇。全市农业转移人口落户城镇2.67万人。

农村面貌不断改善。建成农村公路通畅工程1030公里，行政村通畅率达到68%。新建、续建集中供水工程22处，解决了18.9万农村人口和学校师生饮水不安全问题,贫困地区自来水入户率达到75%。完成靖会、兴电、刘川等5处大型泵站年度更新改造任务，发展有效灌溉面积2.5万亩。兴修梯田14.3万亩，新修高标准基本农田13.6万亩，完成村庄绿化美化4.5万亩，水土保持综合治理148.7平方公里。投入财政奖补资金3000万元，整合项目资金1.2亿元,建成“美丽乡村”示范村10个、环境整洁村138个。

环境治理取得突破。建成白银公司第三冶炼厂废水深度处理回用等4个重金属污染治理和东大沟流域6公里河道综合整治工程，减排重金属废水120万吨；建成工程减排项目15个、规模化畜禽养殖减排项目25个；秸秆饲料化利用率达到60.3%，地膜回收率达到76.3%；完成各类造林32.28万亩；投资1亿元的矿山环境治理工程三期项目开工建设；取缔关停不符合产业政策、污染严重的企业66个，淘汰落后产能353万吨，发展清洁生产企业33户，万元GDP能耗下降到1.51吨标煤；市区空气质量优良天数达到309天。

5.致力保障民生、促进和谐，群众生活日益改善。在财政减收压力大的情况下，各级财政投入改善民生和社会事业资金85.96亿元，占财政支出的75%，比上年提高2个百分点。

民生工程集中实施。省、市确定的32项惠民实事全部办结。落实扶贫专项资金1.77亿元,实施易地扶贫搬迁5335人，5个乡镇46个村实现整体脱贫，减贫人口7万人。新增城镇就业6.72万人，发放小额担保贷款4.6亿元，帮助1.08万失业人员实现再就业，劳务输转30.5万人，创劳务收入44.35亿元。企业退休人员养老金标准调高到月人均1549元，城乡居民社会养老保险基础养老金标准月人均调高至65元，参保率达到96.7%，为30.35万名城乡低保和农村五保供养对象缴纳参合、参保金717.76万元，居民医疗保险政府补助标准提高至年人均320元，建成城乡社区老年人日间照料中心78所，新增养老床位960张。城乡低保标准和补助水平提高15%,发放城乡低保资金5.45亿元；发放农村残疾老人和高龄老人补贴698.7万元，发放孤儿基本生活费1199.8万元，发放残疾人补贴和危房改造资金4289万元，发放城乡医疗救助、临时生活救助资金6746.98万元。争取国开行棚改贷款16.23亿元，开工保障性安居工程6546套，完成农村危房改造9300户。

社会事业加快发展。制定出台了加快教育、卫生改革发展的意见，着力解决资源配置、人员待遇、质量提升等突出问题。新改扩建校舍32.2万平方米，消除危房20.9万平方米，改造寄宿制学校32所、薄弱学校37所,建成会宁、靖远特殊教育学校，新建幼儿园22所，改扩建农村小学附设幼儿园100所,实现了乡镇公办中心幼儿园全覆盖；投资6.5亿元的白银矿冶职业技术学院新校区建成使用，职业教育稳步提升。市第一人民医院住院部开工建设，新改扩建乡镇卫生院14所、标准化村卫生室195个，市儿童医院、市残疾人康复中心主体工程完成;新农合人均筹资标准提高到390元，参合率达到98.04%；为1614例白内障患者免费实施复明手术；为符合条件的372对夫妇落实“单独两孩”生育政策，人口自然增长率5.65‰。建成“乡村舞台”69个、“一村一场”农民体育健身工程69个、乡镇社区体育健身中心6个，开通全省市州级首家移动数字图书馆，平川孝和文化中心被民政部命名为弘扬中华孝道示范基地，成功承办省第十三届运动会、省第九届残疾人运动会暨第三届特奥会。

社会治理不断加强。推进平安白银建设，建立城市大巡防体系，八类严重刑事犯罪案件发案同比下降24%。信访和重大矛盾纠纷化解率、调解成功率达到92.7%和98.7%。深化“法律七进”，推进依法治理，强化法律服务，法律援助办案数量同比增长78%。投资700多万元，建成市地震预警系统。建立领导班子和领导干部安全生产目标责任考核办法，完成县区煤矿兼并重组，关闭低产能小煤矿14处，安全生产形势稳定。开展首届市政府质量奖、标准创新奖评选活动。靖远被命名为省级食品安全示范县。

6.致力转变作风、提高效能，发展环境优化提升。认真开展党的群众路线教育实践活动和“效能风暴”行动，中央“八项规定”、省委“双十条规定”和市委“十六项规定”得到有效落实。会议、文件同比减少22%和21%，“三公”经费下降27%，清理腾退办公用房1.01万平方米。完成各类督查落实事项305件，集中开展了“12+5”专项整治行动，查处“吃空饷”人员99名，处罚违规医务人员12人次，清退不符条件低保对象7045人，查处违反作风纪律规定问题92起，问责处理党员干部238人。人大代表、政协委员建议提案266件全部办复。建立完善重大行政决策集体决策制度，制定了市级财政专项资金、市政府投资项目管理办法，公共资源交易中心受理进场项目1482宗，完成1258宗，交易金额107.68亿元；主动公开各类信息31258条，受理审批服务事项28.22万件，办结率99.8%。梳理制定投资引资优惠政策、优化发展环境暂行办法，严格推行项目帮办、限时办结和并联审批服务，发展环境得到优化。

一年来，审计、统计、双拥、民族宗教、老龄、人防、气象、地方志、公积金、红十字会等工作都取得新的进步。

各位代表，过去一年，经济发展面临前所未有困难，全市人民同舟共济、团结奋进，在改革创新中激发活力，在攻坚克难中赢得发展。特别是面对保增长的巨大压力和促转型的艰巨任务，市委及时研究经济运行、产业发展、民生改善等重大问题，是我们做好工作的坚强领导核心；市人大、市政协积极组织视察调研，提出意见建议，为我们做好各方面工作提供了大力支持；各区县、各部门迎难而上，全力作为，为全市保持平稳发展发挥了决定性作用。在此，我代表市人民政府，向全市广大干部群众、政法干警、驻银解放军和武警消防官兵，向各民主党派、工商联、人民团体，以及所有关心支持白银发展的各界人士，表示衷心的感谢，并致以崇高的敬意！

在肯定成绩的同时，我们也清醒地认识到，我市经济社会发展和政府工作还存在不少困难和问题：一是稳增长的基础还不够牢固，主导产品价格下跌，企业效益下滑，部分重点项目进度不快，经济增速有所回落；二是经济结构仍然不尽合理，三产比重偏低，现代服务业发展滞后，自主创新能力不强，战略性新兴产业处于培育期，中小微企业融资难、融资贵的问题还没得到有效解决，培育新的经济增长点还需下更大功夫；三是农业基础和农村发展条件依然薄弱，城乡公共服务发展水平差距较大，扶贫攻坚和统筹城乡发展难度加大，全面建成小康社会任务艰巨；四是瓶颈制约因素突出，破解交通、水利等基础设施难题还需付出艰苦努力，财政增收、城乡居民增收压力依然很大，社会事业发展与群众的期盼还有差距；五是政府部门效能不高、落实不力、干劲不足的现象还不同程度存在。对这些问题，我们一定要敢于担当、敢于碰硬、敢于创新、敢于争先，以更大的决心、更大的力度、更有效的措施认真加以解决。

2015年主要任务

今年政府工作的总体要求是：深入贯彻落实党的十八大和十八届三中、四中全会及中央、省经济工作会议精神，按照市委七届十四次全委扩大会议部署，主动适应经济发展新常态，坚持稳中求进，坚持改革创新，坚持依法治市，以提高经济发展质量和效益为中心，以兰白科技创新改革试验区、白银工业集中区和丝绸之路经济带白银段“两区一段”为平台，以“3+4”产业集群为抓手，大力实施项目工程，继续开展扶贫攻坚，集中精力推进产业升级、交通提速、园区提质、民生升温，努力实现经济平稳健康发展、社会大局和谐稳定。

经济社会发展主要预期目标是：生产总值增长8.5%以上，工业增加值增长9%以上，固定资产投资增长20%以上，社会消费品零售总额增长12%，公共财政预算收入增长10%，城镇居民人均可支配收入增长10%，农民人均纯收入增长12%，城镇登记失业率控制在4%以内，人口自然增长率控制在7.3‰以内，居民消费价格涨幅控制在3%以内，单位生产总值能耗和主要污染物排放控制在省下达的目标以内。

重点抓好九个方面的工作：

一、推进改革开放，激发发展活力

坚持问题导向、发展导向、市场导向和民生导向，全面落实市委各项改革部署，进一步厘清政府权责边界，充分发挥市场在资源配置中的决定性作用，最大限度释放改革红利。

深化重点领域改革。加快转变政府职能。深化行政审批制度改革，做好“接、放、管”工作，进一步取消、下放市级行政审批事项；建设“三张清单一张网三个平台”，实行限时办理、规范办理、透明办理、网上办理。调整完善政府工作考核评价办法和激励约束机制；深化公共资源交易市场化改革，健全市公共资源交易服务平台和行政监督平台。推进集行政审批、公共服务、效能监察三位一体的市民中心建设，提升便民服务水平；成立不动产登记机构，开展不动产登记工作。全面完成公务用车改革任务。深化财税体制改革。建立地方政府债务管理及风险预警机制，政府性债务全部纳入预算管理，列入发展成果评价体系。完善政府预算体系，改进预算管理控制，建立跨年度预算平衡机制。规范财政专项资金管理，全面实施“三公开”制度，整合存量资金和部门管理的专项资金，采取以奖代补、担保融资等方式提高资金使用效率。清理规范财税优惠政策，全面完成“营改增”任务。深化投融资体制改革。市级政府投资项目实行“代建制”和“双审制”，实现政府投资项目专业化管理。推广实施政府和社会资本合作PPP模式，积极引入社会资本参与存量项目改造和运营，新建市政工程和新型城镇化试点项目优先采用PPP模式建设。建设甘肃股权交易中心白银分中心;设立财政参股的技术创新驱动基金、战略性新兴产业创业投资引导基金、贷款融资担保基金，吸引更多社会资本参与科技创新、结构优化升级等重点领域。创新“三权”抵押方式，建设农村“三权”流转交易中心，建立涉农融资激励制度。加强对小额贷款公司、担保公司、投资公司、典当行的监管，规范资金管理和使用，有效防范风险。深化国资国企改革。积极支持白银公司、稀土公司等国有骨干企业加快股份制改造和直接上市融资步伐；加快推进市城投公司、水务集团、交通投资公司、黄河旅游公司等投融资企业在融资手段、资源资本化、薪酬激励、绩效考核等方面改革创新，推动供热、供水、公交等公益性企业产权和投资主体多元化。推进资源性产品价格改革。建立居民生活用气阶梯价格制度，推进供水、供热、供气等“一户一表”改造，实行终端收费。推进农村产权改革。开展农村土地承包经营权有序流转、农村土地征收、集体经营性建设用地入市、宅基地制度改革，基本完成宅基地和集体建设用地确权登记发证，在20个乡镇开展农村土地承包经营权确权登记颁证工作。加快教育卫生体制改革。加强依法治教、依法治校，推进教育“管办评”分离;全面推行义务教育阶段学校校长职级制;推进校长教师交流，到2017年实现县域内交流制度化、常态化。全面开展县级公立医院管理体制改革，完善绩效考核制度，逐步建立法人治理结构；在白银区开展“城乡医疗一体化”管理改革试点；完成县区卫生计生委组建。

建设丝绸之路经济带明星节点城市。对接国家“一带一路”战略规划和全省总体建设方案，争取将我市重点铁路、公路交通项目纳入互联互通工程；对接能源战略通道，加快推进复合型能源基地建设；通过承接产业转移、引进有实力的企业共同建设生产加工出口基地；加强与中亚国家开发合作，在文化交流、旅游联动、农业协作、园区共建、商贸流通等方面实现突破。加强与兰州新区等周边地区的沟通协作，在要素互融互通、资源共享上取得成效。

二、保障改善民生，维护和谐稳定

坚持守住底线、突出重点、完善制度，更加注重保障基本民生，更加关注低收入群众生活，更加重视社会大局稳定，让发展成果更多更公平惠及全市人民。

抓好发展型民生。加大精准扶贫力度，落实项目审批和资金整合“双到县”政策，加大行业部门扶贫攻坚力度，新建改建各类农村公路600公里，年底通畅率达80%以上；引导贫困片区人口出山入川、出村入镇、出乡入城，实施易地搬迁项目，搬迁2194人；解决22.66万农村人口和学校师生饮水不安全问题，贫困地区自来水入户率达到80%以上；完成贫困户“两后生”培训2万人，实现全市扶贫对象年均纯收入增幅高于全市平均水平2个百分点，减少贫困人口5万人。全面开展大众创业，完善创业政策体系，加强创业孵化基地建设，启动高校毕业生创业引领计划，健全职业介绍、就业指导等公共就业服务体系和服务平台，推行就业援助对象实名制管理服务，确保城镇新增就业6.2万人以上。完善企业、机关、事业单位工资增长机制，推进企业工资集体协商制度，在县以下机关实施职务与职级并行、职级与待遇挂钩制度试点。

抓好保障型民生。实行善政养民，健全完善惠农政策信息公开网，做到民生资金“四级公示”、“一卡通行”，农民工子女“零障碍”入学。城市、农村低保保障标准分别提高10%、11%；农村五保供养补助标准提高40%；采取分档资助方式，为所有城乡低保、农村五保供养对象缴纳2016年参保参合金。落实中等职业教育全部免学费政策，使每一个贫困家庭孩子都能够接受中职教育；实施“全民参保登记计划”，建立健全兼顾各类人员的社会保障待遇确定和正常调整机制，推进居民医疗保险城乡统筹整合，推动异地参保人员医疗保险费用实时结算，实现网上“一站式”服务。健全临时救助制度，做好“救急难”工作，提升受灾群众救助能力。加快残疾人社区康复示范点建设，新建社区康复服务站10个。发放廉租住房补贴7529户，实施农村危房改造1万户，住房保障逐步转向实物保障和货币补贴并举，推进棚户区改造货币化安置，用政府购买服务等方式把符合条件的存量房转为公租房。

抓好普惠型民生。优先发展教育事业，大力促进义务教育均衡发展，继续实施好“全面改薄”项目，消除危房10万平方米，改扩建校舍12万平方米，建设标准化学校100所；优化配置县域义务教育资源，加快教育信息化建设，加快普通高中优质特色发展，大力发展以就业为导向的现代职业教育。深化产教融合和校企合作，完成市工业学校与白银职业中等专业学校资源整合，加快职业教育集团化，推进中高职一体化办学。公开招考幼儿教师150名、特殊教育教师10名，认真落实乡村教师补助政策，提高班主任津贴，努力改善教师待遇。办好白银矿冶职业技术学院，推动靖远师范与兰州城市学院联合办学。大力发展卫生事业，积极建立惠及农村的远程会诊系统，加快标准化村卫生室、乡镇卫生院建设，实现标准化村卫生室全覆盖；完成市第一人民医院住院部综合楼主体工程，市儿童医院、省中医院制剂中心全面完工并投入使用，推进市第三人民医院（会宁）建设。新农合平均参合率保持在97%以上，筹资标准从390元提高到450元。继续推进计划生育利益导向示范区创建工作，贯彻执行“单独两孩”生育政策。积极发展体育事业，建成乡镇社区体育健身中心6个。办好全市青少年田径运动会、第四届职工运动会。

依法加强社会治理。落实国务院《社会信用体系建设规划纲要》，全面启动社会信用体系建设，建设信用记录采集系统和市县信用信息共享平台，建立社会诚信、法院执行、安全生产、产品质量等红黑榜名单和惩戒措施，推进政务诚信、商务诚信、社会诚信和司法公信。深入推进警务运行机制改革，开展校园安全、街面防范、道路畅通、服务提升、警务公开等“五大行动”，完善立体化社会治安防控体系。做好“六五”普法总结验收工作，深入开展法治城市、法治县区、法治学校、民主法治示范村、学法用法示范机关（单位）、诚信守法企业创建活动。落实诉访分离制度，打造“阳光信访”。加强双拥工作，建设“铜城英烈纪念碑”。建设市防震减灾科普宣传教育基地，创建国家防震减灾示范城市。深入宣传落实新《安全生产法》，健全市县乡村安全生产责任体系，建设全省安全生产应急救援平台白银基站和全市安全生产在线监控系统。开展食品安全示范城市、示范县创建工作，建成市食品检验检测中心，全面提升食药安全保障水平。

集中力量办好九件惠民实事。（1）实施整村推进50个；（2）建成“乡村舞台”135个；（3）改扩建农村小学附设幼儿园40个；（4）建设“一村一场”农民体育健身工程100个；(5)补助法律援助办案经费100万，为更多困难群众提供法律援助服务；(6)建设便民连锁服务店100个；(7)改造老城区失管小区30万平方米；(8)建设市区和各县城公共厕所45座；(9)培训红十字应急救护人员2.5万人。

三、调整产业结构，推动提质增效

推进农业现代化。深入实施“358”现代农业发展行动计划,落实最严格的耕地保护制度，突出发展玉米、马铃薯两大作物，种植面积分别达到140万亩、100万亩以上，粮食总产量稳定在76万吨以上，确保粮食安全；推广全膜双垄沟播旱作农业138万亩，推广高效农艺节水114万亩，创建高产万亩示范片20个。新改扩建日光温室1万亩、塑料大棚6000亩，实施好靖远北方城市冬季设施蔬菜种植基地试点县项目，瓜菜种植稳定在100万亩以上；新增枸杞、甘草、党参等中药材2万亩，抓好靖远中药材大县基地建设；加快先进实用农机具推广应用，扶持发展农机专业合作社30个，推进农机农艺融合，提高农业现代化装备水平。深入实施草食畜牧业发展行动，促进粮经饲种植结构协调发展，新增标准化规模养殖小区70个，牛、羊饲养量分别达到17.5万头、485万只；建设优质林果基地15万亩。建立白银“三农”数据库，完善农业经营体系，新增龙头企业20家,创建市级以上农民专业合作社300家、家庭农场50家；发展农村电子商务，推动农业“接二连三”，扶持乳制品加工项目，农产品加工转化率达到52%；引导规范农村土地有序流转,流转面积达到110万亩，实现县区土地流转服务中心和仲裁庭全覆盖；加强农产品质量安全监管，突出特色农产品品牌建设，扩大“三品一标”覆盖面，创建农业部标准示范园3个，省级标准化示范小区20个。

推进工业新型化。加快老工业基地调整改造步伐，不断延伸“3+4”产业链条，全年工业投资增长25%。全力改造提升传统产业。完成白银公司铜冶炼技术提升改造、锌冶炼综合利用等项目；积极协调推进华鹭铝业出城入园深加工、银光公司2万吨AKD、中核集团20万吨钛白粉、刘化集团硝基复合肥、液体硝铵和煤气化、甘肃华盛45万吨新工艺芳烃、重庆昌元10万吨重铬酸钠及配套清洁生产、中天高纯碳酸锂及高纯氟化锂等项目；加快推进热电联产、魏家地煤矿改扩建项目。实施平川紫砂土资源综合开发，新建雄立紫砂生产线，打造西部特色紫砂产业链条。大力发展战略性新兴产业。新材料产业实施白银公司高精度电子铜带、稀土公司高性能抛光粉、宏达精铝产品开发、大河稀土凹凸棒精细加工、郝氏炭纤维原丝配套等项目，争取郝氏炭纤维公司列入全省战新产业总体攻坚战第二批骨干企业；新能源产业实施天海镍氢动力电容电池项目，国电靖远东升贺寨柯、会宁华家岭等5个风电项目，中电景泰等3个光伏发电项目，新增风电装机30万千瓦，光电装机10万千瓦，分布式光伏发电5万千瓦，打造山地风光电新能源基地；生物医药产业实施杰康诺谷胱甘肽高技术产业化、力华生物有机肥、熙瑞菊粉加工扩建、陇草堂中药饮片等项目，延伸拓展生物医药、生物制品、生物育种等高附加值产业链；节能环保产业实施启明星照明节能产品、甘肃亿维节能变压器等项目，推进生活垃圾焚烧发电、废旧汽车拆解利用、“双百工程”示范基地建设；装备制造产业实施智能制造试点示范专项行动，开展神龙航空、荣信科技等10家装备制造企业“两化”融合试点；信息产业加快推进数云白银云计算产业园建设，实施一批信息惠民工程，引进国内IT企业参与项目建设运营，推进智慧城市、信息惠民、信息消费试点城市建设。战略性新兴产业增加值增速达到16%以上。着力强化企业服务。建设中小企业公共服务体系，建立市级综合服务窗口平台，在县区和工业园区设立服务中心。加强对60户产值过亿元企业生产经营情况动态监测分析，协调煤电运油气等生产要素保障，落实差别电价和燃煤电厂脱硫电价政策。建设省级陶瓷检验中心、甘肃中部槽车检验中心，组建质量检验检测工程公司。加大商标培育力度，推动“敦煌”牌、“银光”牌商标争创中国驰名商标。

推进服务业现代化。加快物联网、信息产业、金融、现代物流等生产性服务业发展，重点培育3家省级、15家市级生产性服务业企业，服务业增加值占比提高1个百分点以上。大力发展商贸物流业。做好会宁供销社综合改革试点工作，健全市、县、乡、村四级网络体系；实施农耕食文化活态传承工程，建设会宁小杂粮和景泰蜂蜜文化产业园。加快银西商贸和银东综合两个核心物流园建设进度，力争纳入省沿边地区开发开放规划。协调推进由白银公司牵头，银光公司、靖煤公司参与组建的白银铁路物流公司，加快资产重组，建设保税物流中心。重点培育商品专业交易市场5个、城市商贸综合体5个；实施万村千乡市场升级工程，把农家店建设成为电子商务网下店；加快推进长通路益民市场、长安路综合农贸市场、雒家滩旧货市场、新天地文化商业街等项目建设，建设“放心粮油”示范店15个。繁荣活跃现代金融业。实施金融惠民工程，加快发展村镇银行和农村资金互助组织，实现地方银行县区分支机构、县区村镇银行、贫困村农民资金互助组织、乡镇金融服务站全覆盖。扩大信贷规模，优化信贷结构，确保各项贷款增长18%以上，其中，中小微企业贷款和涉农贷款增长20%。支持金轮股份、伟慈药业、中核钛白增资扩股和西藏矿业定向增发，引导和支持中小微企业通过集合债、“新三板”、私募债等融资模式直接融资。大力发展现代保险业，积极开展中小微企业、农户小额等保证保险贷款业务。扩大城市建设投融资公司融资规模，加快组建县区园区投资、交通投资和困难农户贷款政策性融资担保公司，担保贷款增长20%。加快发展健康养老业。开工建设四龙养老服务中心，推进城乡社区日间照料中心建设,建立居家养老服务信息网络和服务平台；依托省中医院制剂中心，规划建设中医文化街及中医馆；推进会宁西岩山中医药养生旅游生态园建设。积极发展电子商务。依托甘肃邦农现代农贸物流中心，建设电子商务园区；加快实施靖远县与阿里巴巴“千县万村”电商工程合作、会宁县智能化电子商务平台等项目，建设“白银特色商品展示馆”。

发展壮大非公经济。建立健全许可审批与后续监管相衔接的市场监管责任机制，进一步削减资质认定项目，放宽注册资本登记条件，积极探索推行电子营业执照。培育发展“四上”企业52家，文化产业法人单位30家；加快中小微企业服务体系建设，开通中小企业公共服务平台和维权投诉中心，全面推行负面清单，落实非公经济进入特许经营领域政策措施，支持民间投资加快进入能源、市政、保障性安居工程等领域。

四、抓好项目工程，增强发展后劲

全力建设项目。落实重大项目并联审批和帮办服务，推进50个市列重点项目完成年度投资160亿元，全市固定资产投资突破500亿元。交通运输方面，开工建设景泰至中川高速公路、国道247线靖远至会宁段改造、国道312线会宁段改造、包兰铁路扩能改造银川至兰州客运专线、白银通航机场、银东客运货运枢纽中心等工程；加快推进白银至中川机场快速通道、省道217线白银至景泰二级公路改扩建、干塘至武威南增建二线白银段建设等项目建设进度。水利工程方面，开工建设黄河干流白银段防洪治理、会宁县祖厉河城区段综合治理二期工程，重点推进引洮一期会宁北部供水、靖远双永供水、兴电灌区齐家大岘隧洞除险加固、靖会干渠甘沟延长段暨会宁城区应急供水、景泰黄崖坝综合开发、河西走廊国家级百万亩高效节水示范、大型泵站改造等项目，发展有效灌溉面积2万亩，兴修梯田12万亩，完成水土保持治理面积140平方公里。

努力争取项目。积极对接国家、省规划和政策投向，健全完善项目谋划和入库管理责任制，确保“3341”项目入库保持在1850个以上，总投资达到3500亿元以上。重点做好银光公司新建10万吨TDI、靖煤集团低热值煤发电、国电靖远发电“上大压小”、黑山峡梯级开发、环海中铁路、白银至平凉铁路、兰州至景泰龙湾沿黄二级公路、国道247线景泰至平川段、国道341线平川至白银段、打拉池瑞兰通用航空产业基地、引洮二期、甘肃中部生态移民扶贫开发供水、靖远金滩黄河大桥等重大项目前期工作，争取早日开工建设。

着力引进项目。坚持产业链招商，围绕“3+4”产业集群，完善市级领导联系重点招商项目制度，加快引进一批符合产业导向、附加值高、财税贡献大、带动能力强的优势项目。严格落实“帮办制”，实行招商引资项目行政审批服务“一卡通”，对已签约重点招商项目靠实具体帮办责任人，落实引资单位、职能部门、园区、服务考核中心“四位一体”帮办服务体系，打造“白银帮办”全省服务品牌。落实好促进引资和投资优惠、招商引资中介人奖励办法等激励政策，严格兑现土地、财税等扶持政策，营造良好投资环境。实现招商引资到位资金增长率达到20%以上，项目落地率50%以上。

扎实做好“十三五”规划。根据国家、省“十三五”总体规划基本思路和市委编制“十三五”规划要求，加强前期课题研究，科学编制总体规划纲要，探索国民经济和社会发展、城市总体规划、土地利用规划“三规合一”。研究确定一批带动作用强、事关全市长远发展的重大基础设施、产业投资项目，并争取更多纳入国家和省“十三五”发展规划。

五、实施创新驱动，推进园区开发

提升创新能力。建设兰白科技创新改革试验区，以高新区为主体，创新试验区管理体制，争取国家差别化政策。积极施行中关村先行先试股权激励、科技成果使用处置、收益管理等6项政策。设立兰白科技创新改革试验区建设专项资金。加大科技孵化器一期、二期招孵力度,入孵企业达到120家，企业授权专利增加到300件，把孵化器建设成为西部一流的国家级孵化平台；各园区引入社会资本分别建设2万平方米以上孵化器。推进全国知识产权试点城市建设，完善产学研协同创新机制，提高企业自主创新能力，研发省级新产品和新技术15个，培育省级企业技术中心2个、企业研究院1家。开展“专利工作提升年”活动，万人发明专利拥有量达到1.5件。完善科技成果转移转化服务体系，重点推进白银新材料研究院、兰州大学白银技术转移中心筹建工作，支持上海杨浦兰白创新改革试验区技术转移中心开展工作，推动高校、科研院所科技成果在银转化，实现科技成果资本化、产业化。开展白银籍在外知名人士“归根”工程。

提升承载能力。高新区全力推进银东工业园管网改造等工程；银西工业园完成园区道路、给排水、供电等工程；刘川工业园以建设省级工业园为目标，加快推进亚行二期贷款建设项目，完成工业大道等5条主次干道路基、污水处理厂及配套管网、综合服务区、湿地景观带等工程；平川工业园完成南区路网、电网、绿化等工程；正路工业园完成园区调蓄水库、净水厂、污水处理厂、二期道路等工程；会宁工业园完成西城兴业路、郭城南片区道路及给排水等工程。白银工业集中区实施基础设施项目42项，总投资突破111.06亿元，年度完成19.51亿元。

提升集聚能力。推进6个“园中园”集聚发展。银西工业园建设生物医药园，引进国内外知名生物医药产业化企业，研究培育生物医药领域优势技术和优势产品。刘川工业园建设炭纤维产业园，重点延长从原丝到炭绳、炭毡、炭炭复合材料的产业链条；建设稀土新材料园，发展稀土新材料应用和深加工；建设光热装备制造园，推进深圳前海金阳光、富源科技、爱能森科技塔式太阳能热电站和装备制造基地项目。银东工业园建设精细化工园，围绕银光集团配套上下游产业，推进重铬酸钠、钛白粉、芳烃和异氰酸酯深加工系列产品等项目。高新区建设新能源装备产业园，重点推进电容电池、碳酸锂等项目。加快白银工业集中区创新集聚发展，工业增加值增长11%以上。

六、推动文化体育旅游融合发展

认真落实《关于加快推进文化体育旅游融合发展的意见》，全年文化产业增加值增长25%以上。

加快发展文化事业。筹备做好庆祝白银恢复建市30周年和会宁会师暨长征胜利80周年工作，实施文艺创作“五个一”工程，拍摄《大会师》、《岳钟琪》等影视作品，实施数字图书馆推广工程。组建民间文化社团200个。举办第十五届PHE国际青少年儿童书画大赛、“丝路?黄河”全国美术作品展和第二届民间收藏书画交流活动。推进市文化艺术中心、国际青少年儿童美术博览馆等项目建设，加快实施永泰城址保护维修工程，推进龙湾古镇建设。

大力发展文化体育旅游产业。在公共服务体系建设中注重文化、体育、旅游项目功能设计和融合。全力办好黄河石林文化旅游节、会宁红色旅游文化节、金凤凰少儿艺术节等节会赛事。打通靖远县城至平川黄湾沿黄快速通道，建设30公里黄河风情精品线。加快黄河石林管理体制改革，组建黄河石林开发总公司，争创5A级景区；整合会师圣地文化旅游资源，创建全国知名旅游品牌。大力开发民俗工艺特色旅游产品，繁荣古玩与艺术品交易市场。

加紧建设文化体育产业园区。推进会宁红色文化产业园、靖远南华山生态文化产业园、景泰黄河石林文化产业园、白银区铜工艺产业园、黄河假日城、平川艺术陶瓷产业园建设，大力开展文化招商，吸引旅游开发、文化创意、艺术品加工骨干企业入驻园区发展。设立创业奖励基金，扶持各类人才来园区创业投资。开发体育旅游项目，完善提升靖远腾飞国际赛车场、滑雪场运营水平，规划建设山地体育公园，打造体育旅游经典项目品牌赛事。

七、加快新型城镇化建设

坚持城乡统筹、城乡同治，推动城镇公交、电力、供水、通讯、消防等基础设施向农村延伸，城镇化率提高1.5个百分点。

加强中心城市建设管理。深入开展“五城联创、六城同建”活动。对接国家、省新型城镇化发展规划和“三个1亿人”城镇化实施方案，编制新型城镇化规划、11个片区棚户区修建性详规，修编白银城区基础设施专项规划，完成沿黄新区控制性详规，推进市县“多规合一”。加大信息化建设力度，实施农村光网工程；构建城乡规划信息化管理平台，完善地下管线信息系统。新建上海路北段、凤凰街、北环路西延段、水川路、银山路道路5条，续建西山路、北环路、银西中路等道路6条，打通市区环路。实施金岭公园城区防洪及综合治理、高速公路西出口绿化工程，启动长通公园改造、上下孤山森林公园建设工程，美化城区环境。加快市区供水、污水、热电联产配套管网、银西工业园区热源厂基础设施建设和老旧供热管网改造工程，完善城市功能。全面推进中心街、八号楼等片区和刘家梁、大井子、黄茂井城中村改造，新建保障性安居工程5951套，改造棚户区4478户。重点推进工程质量治理行动，严厉打击建筑施工挂靠、转包和违法分包行为，提升工程质量。

提升县城和重点镇综合承载能力。依据自然环境和历史文化禀赋开展特色化城镇建设，统筹用好全市小城镇发展专项资金，整合其它各类资金，加快推进靖远县、郭城驿镇、四龙镇“一县两镇”新型城镇化省级试点和6个市级示范镇建设。重点实施会宁会师大道一期工程，靖远南滨河路景观带、怡园古城，景泰寺滩沙河城防工程、世行贷款红水镇基础工程，白银城区109国道两侧改造美化，平川环城北路、宝水快速通道等城市建设重点工程。做好双龙乡等历史文化名镇名村保护申报工作。

推进农业转移人口市民化。取消暂住证，推行流动人口居住证制度。搭建进城务工人员“培训—就业—维权”一体化服务平台，开展转移人口就业技能培训。探索建立财政转移支付、城镇建设用地增加与农业转移人口市民化挂钩机制，做好农村与城镇之间养老保险、医疗保险等险种衔接，把进城落户农民纳入社会保障体系，给予“城乡兼有”身份待遇。

八、推进生态文明建设

坚持循环发展。深入开展全省“457”循环经济推进行动，大力实施清洁生产、固体废物综合利用、农村能源建设、种养殖废弃物综合利用等项目，强化公共机构节能管理，建设再生资源回收利用体系。高新区全面完成循环化改造，白银西区、平川中区完成60%以上循环化改造任务。以推行企业清洁生产为重点，完成全市35户骨干企业建设任务。申报国家循环经济示范城市和“城市矿产”示范基地。

加强环境保护。深入开展环境保护大检查，推进工程减排，抓好白银公司3个重金属污染治理、国电靖远发电、铜城热力公司脱硫脱硝工程，实施东大沟土地重金属污染综合整治项目，力争把我市建成国家重金属污染治理示范市；建成甘肃稀土新材料废水深度治理项目，加快建设白银区污水处理再生利用工程，完成10家规模化畜禽养殖场和养殖小区污染治理工程。实施西北铅锌冶炼厂燃煤锅炉升级改造、余热余压利用等重点项目，加快城区燃煤锅炉改造和淘汰步伐，做好市区茶浴炉清洁能源替换和供热小锅炉并网拆除工作；推进平川、靖远热电联产集中供热工程，逐步实现城市供热集中化。实行强制性清洁生产审核，加强重点排污企业监管。启动PM2.5空气质量监测，确保优良天数按新标准达到190天以上。

改善城乡环境。开展城市园林化、道路景观化、家园清洁化行动，城市绿化重点实施天津路、长安路、金岭公园、金鱼公园提质改造，创建一批园林化单位；道路景观重点抓好城市出入口道路绿化美化工作，推进环乡村景观林带建设；家园清洁化重点整治垃圾乱倒、道路乱挖、车辆乱停、摊位乱摆等问题。新建“美丽乡村”示范村10个和环境整洁村138个，切实改善农村人居环境。

加强生态建养。认真落实国家生态安全屏障综合试验区建设任务，严格执行封山禁牧，启动实施新一轮退耕还林工程，完成村屯绿化、农田林网、林缘区造林、城区大环境绿化等生态林业17万亩，建设核桃、枸杞、大枣等为重点的民生林业15万亩。全面推进集体林权制度综合配套改革，加快林地流转，发展以种植养殖为主的林下经济。

九、加强法治政府建设

认真开展“工作落实年”活动，把法治思维和法治方式贯穿于政府工作全过程，严格执行市委决策，自觉接受人大的法律监督和工作监督、政协的民主监督。完善依法决策程序和制度，把公众参与、专家论证、风险评估、合法性审查、集体讨论决定确定为重大行政决策的法定程序，建立重大决策终身责任追究制度及责任倒查机制，强化决策后的跟踪反馈和纠错问责。设立政府法律专家咨询委员会，建立政府法律顾问制度，实现市、县、乡三级政府全覆盖。深化行政执法体制改革，严格规范公正文明执法。强化OA协同系统办公，规范公文运转和审批决策流程，推进财政预算、公共资源配置、重大项目、社会公益事业等领域政府信息公开常态化，确保政府权力在阳光下运行。严格落实党风廉政建设主体责任，健全廉政风险防控体系，强化源头治理和制度防腐，科学编制财政预算，从严控制“三公”支出，从严落实预算执行；加强行政审批、项目招投标、土地出让、政府采购等重点领域的行政监察和审计监督。加强公务员队伍建设，强化行政效能监督问责，加大明察暗访力度，持续改进工作作风，大力提升机关效能，不断提高工作水平、办事效率和服务质量。

各位代表，站在历史新起点，面对人民新期待，我们倍感使命光荣、责任重大；倍感机遇难得、时不我待。让我们在市委的坚强领导下，坚定信心、锐意改革，开拓创新、奋发有为，为建设经济发展、社会和谐、环境优美、文明幸福的新白银而努力奋斗！

术 语 解 释

“万吨农产品进京”：市政府以“农超对接”、“农校对接”为主要途径，与北京物美、华联、京客隆、华润万家、沃尔玛、家乐福等多家超市建立了业务联系，在我市建立了两处“北京物美、华联集团西瓜枸杞直供基地”，签订了6000吨农产品供货协议；建立了两处首都高校食堂蔬菜直供基地，签订了4000吨农产品供货协议，为本地名优农产品找到了一条畅通的销售之路。

阿里巴巴“千县万村”计划：阿里巴巴集团计划于今后3至5年内，投资100亿元在全国建立1000个县级电子商务运营中心和10万个村级电子商务服务站。

“新网工程”：为健全农村市场体系，发展适应现代农业要求的流通产业，全国供销合作总社决定实施“新农村现代流通服务网络工程”（简称“新网工程”），旨在大力发展农业生产资料现代经营服务网络、农副产品市场购销网络、日用消费品现代经营网络和再生资源回收利用网络。

“三权”：即农村土地承包经营权、宅基地使用权和林权。

“营改增”：即营业税改增值税。2011年，经国务院批准，财政部、国家税务总局联合下发试点方案。截止2013年8月，“营改增”范围已推广到全国试行。

兰白科技创新改革试验区：2014年8月，国务院总理李克强在兰州主持召开促进西部发展和扶贫工作座谈会时强调，东部地区已经建立了若干科技创新的试验区，西部地区也可以搞试验。同年12月，科技部同意支持我省依托兰州新区、兰州高新技术产业开发区、白银高新技术产业开发区、兰州经济技术开发区开展兰白科技创新改革试验区建设试点，并要求兰白试验区要深入实施创新驱动发展战略，以深化科技体制改革为主线，以提高区域创新能力为核心，着力营造良好创新环境，推动产业优化升级，打造丝绸之路经济带重要节点，积极探索西部欠发达地区依靠创新驱动、实现跨越发展的新模式。

“五城联创、六城同建”活动：我市精神文明建设工作以全国文明城市创建为龙头，全面开展“五城联创”（全国文明城市、全国双拥模范城市、国家卫生城市、国家环保模范城市、省级园林城市）和“六城同建”（厚德之城、礼仪之城、诚信之城、洁净之城、秩序之城、书香之城）活动，群策群力建设文明白银。

“单独两孩”生育政策：指一方是独生子女的夫妇可以生育两个孩子的政策。

“乡村舞台”：指在整合我省农村现有文化资源的基础上，利用乡镇文化站、村社文化室、农家书屋、乡村体育健身工程等阵地，组建村级民间自办文化社团，搭建群众自娱自乐的综合性文化服务中心。

“12+5”专项整治行动：我市把专项整治工作作为狠刹“四风”问题的有效举措，集中开展了针对文山会海、庸懒散乱、“吃空饷”等问题以及公正执法、惠农政策落实等12个方面内容的专项整治行动。中央和省委部署有关专项整治任务后，我市又把规范城乡低保、扶贫资金监管、整顿软弱涣散党组织、“会所中的歪风”、“形象工程”和“政绩工程”等5个方面的内容纳入专项整治范围。

“三张清单一张网三个平台”：指政府部门权力清单、责任清单、财政专项资金管理清单和政务服务网，打造网上办事的并联审批平台、快捷务实的便民服务平台、信息公开的阳光政务平台。

“三公开”：即财政预决算、部门预决算和“三公”经费预决算公开。

“代建制”和“双审制”：代建制,是指由市政府投资项目代建管理办公室代表市政府行使出资人职责，对政府投资建设的非经营性项目，通过招标方式选择专业化的项目管理公司负责建设阶段实施，严格控制项目的规模、投资概算、使用功能及质量、安全和工期，项目建成并竣工验收合格后移交给项目业主单位的制度。双审制,是指审计部门依法对代建项目投资概算执行情况、竣工决算情况进行审计监督。

PPP模式：即公私合作模式，是公共基础设施一种项目融资模式。在该模式下，鼓励私营企业与政府进行合作，参与公共基础设施的建设。通过这种合作方式，合作各方可以达到与预期单独行动相比更为有利的结果。合作各方参与某个项目时，政府并不是把项目的责任全部转移给私营企业，而是由参与合作的各方共同承担责任和融资风险。

“358”现代农业发展行动计划：即壮大提高瓜菜、草畜、林果三大特色优势产业；建设以大坝、大坪为核心的省级高效设施农业示范区、以高扬程灌区为重点的节水高效农业示范区、以全膜双垄沟播技术为主的旱作农业示范区、以无公害为目标的优质农产品示范区、以会宁为主要区域的现代草产业开发示范区等五大高效农业示范区；大力实施基本农田建设工程、农业设施装备提升工程、农业科技创新工程、重大农业增产增效工程、农业产业化提升工程、农业公共服务能力提升工程、新型职业农民培育工程、农业经营体制机制创新工程等八大农业提质增效工程。

农耕食文化活态传承工程：该工程是全省供销社系统贯彻省委、省政府建设华夏文明传承创新区的重要举措和实际行动，是一项涉及文化、农业、旅游、供销等多个产业融合发展的系统性工程。工程建设《规划》核心是建设甘肃农耕食文化综合体、建立甘肃农耕食文化传承体系、搭建甘肃特色农产品电子商务平台、培育新型农村市场主体、提升农业科技服务水平、构建农特产品质量安全溯源公共服务平台。

“四上”企业：规模以上工业企业、资质等级建筑业企业（房地产开发经营企业）、限额以上批零住餐企业、规模以上服务业企业等四类企业的统称。

“457”循环经济推进行动：即加快构建循环型工业、农业、服务业和社会“四位一体”体系，扎实推进基地、园区、产业链、企业和项目“五大载体”建设，示范推广区域发展金昌模式、工业企业白银公司模式、园区天水高新农业模式、张掖农业模式、节水型工农业复合定西模式、煤炭资源综合利用窑街煤电模式、城市餐厨垃圾资源再生利用兰州模式等“七大特色模式”。

新标准监测空气质量：2012年2月，新《环境空气质量标准》（以下简称《标准》）正式发布，省会城市成为首批实施环境空气质量新标准的城市。我市今年１月１日起正式开展空气质量新标准监测。新标准监测相比于老标准，由原来的二氧化硫(SO2)、二氧化氮(NO2)和可吸入颗粒物(PM10)三项指标增加到六项，即增加了细颗粒物(PM2.5)、一氧化碳和臭氧8小时浓度限值监测指标，监测指标更全面，评价体系更完善，与人体感官更一致。
